# Supplementary material for: Time scales and gaps, Haar fluctuations and multifractal geochronologies
Source: Commun Earth Environ. 2026 Jan 29;7(1):208. doi: 10.1038/s43247-026-03226-3 (PMC12956591; doi:10.1038/s43247-026-03226-3)
Supplement: Supplementary file 2 — Time scales and gaps, Haar Fluctuations and multifractal Geochronologies Supplementary Material [file 43247_2026_3226_MOESM2_ESM.docx]

# Time scales and gaps: Haar Fluctuations and multifractal Geochronologies

Shaun Lovejoy^a^, Rhisiart Davies^a^ , Andrej Spiridonov^b^, Raphael Hebert^c^, Fabrice Lambert^d^

^a^ Physics, McGill University, 3600 University St., Montreal, Quebec, H2A 3T8, Canada.

^b^ Department of Geology and Mineralogy, Faculty of Chemistry and Geosciences, Vilnius University, M. K. Čiurlionio g. 21/27, Vilnius 03101, Lithuania.

^c^ Alfred-Wegener Institute Helmholtz Centre for Polar and Marine Research, Telegrafenberg A45, 14473 Potsdam, Germany.

^d^ Geography Institute, Pontificia Universitad Catolica de Chile, Vicuña Mackenna 4860, Santiago, Chile.

# Supplementary Material

# Table of contents:

Part 1:  The Isson δ^18^O Carbonate assemblage: 0 - 3.504 Gyrs:

Part 2: Full analyses

Estimates for datasets in the main text

Holocene analyses

Quaternary analyses

Removal of large timescale trends

This supplementary material includes a detailed discussion of the Precambrian series presented in fig. 3 (part 1) as well as the remaining analyses that were not included in the main paper due to space limitations (part 2). The details of data sets and references to the original sources are given in tables 1, 2.

## Part 1: The Isson ^18^O Carbonate assemblage: 0 - 3.504 Gyrs

Over the Holocene and Quaternary we analysed numerous series from individual cores, some were highlighted in the main text (figs 2 - 4), others are analyzed in part 2 of this supplement. These cores enabled us to explore time scales Δ*t* up to several hundred kyrs (the ice cores). For larger Δ*t*, we need older and longer chronologies such as the Haq sea level proxy, or the Grossman benthic stack, both of which give information on statistics of Δ*t* up to the order of hundreds of millions of years and both of which were produced by merging data from multiple locations, effectively filling in some (but by no means all!) gaps. Indeed, it is these merged products that enable us to statistically characterize the largest Δ*t*, yet geochronologies from these merged products may have somewhat different measurement statistics than those from single cores.

For obvious reasons, there is particular interest in time scales comparable to the age of the Earth; in fig. 3 we showed analyses from the Precambrian Isson^1^ δ^18^O Carbonate series that goes back 3.504 Gyrs. It was mentionned that the chronology was extremely heterogeneous, in this part 1 we scrutinize this further.

Fig. S1 shows both the strongly spiky and clustered measurement density ρ(*t*) as well as the δ^18^O Carbonate values discretized at 100 kyr resolution. Beyond the strong spikiness (similar to that the Grossman^2^ stack, fig.1) we see that there is a strong concentration of measurements, 81% were from Phanerozoic (here taken as <600Myrs). The mean densities were: Phanerozoic 34.5/Myrs; Precambrian 1.6/Myrs; full range 7.4/Myrs. Also, at this 100 kyr discretization, 2326 of the 35040 intervals had one or more measurements. The choice of 100 kyrs was somewhat arbitrary since the actual nominal resolutions varied greatly according to the age see fig. S2 for more details.  It should be underlined that the Haar fluctuations work by first averaging and then differencing so that they effectively systematically degrade the resolutions so that the nominal resolution is really just the highest resolution (smallest Δ*t*) that can be meaningfully used.  If the nominal resolution is artificially short, as long as it is more or less fixed (constant) throughout the length of the chronology, it is sufficient to simply ignore the statistics from the shortest Δ*t*. The real difficulty comes when the nominal resolution is highly variable over the length of the chronology. This is case in the Precambrian Isson chronology, which is very short for young sections of the chronology and much longer for the older sections. It turns out that Isson is the only chronology that we analyzed where this is a problem, we investigate it below.

A rough way of estimating the nominal resolution of the measurements is to plot the nonzero intervals Dt between successive measurements (fig. S2). Although this is a highly variable scatterplot, the minimum nonzero difference is the nominal resolution: it was presumably singled out by the scientists who took the data as being an approximation to the actual resolution. For example, in this log *t* - log Dt plot, we see that there are age ranges with resolutions as small as 100 years; we can see this duration (indicated with the bottom dashed line) along with several integer multiples evident as horizontal lines of points above it. In somewhat older sections, it increases to 10 kyrs (the upper dashed line), later (most of the Precambrian) to 1 Myr. Also plotted is the corresponding plot for the Grossman stack (red). We see that, as claimed by the authors, the resolution of 10 kyr is respected throughout. More details are given in the figure caption.

In order to see if there are significant differences in the density and δ^18^O statistics between the Phanerozoic and Precambrian, they are analyzed separately in fig. S3 and S4. While the mean densities differ by a factor of 22, after normalization by the mean,
 $\left. <\Delta\rho\right.>/\overline{\rho}$ only differ by a factor ≈ 2 (fig. S3). We see that up to Δ*t* ≈ 40 Myrs, the shapes of Phanerozoic and Precambrian curves are both quite flat (*H* ≈ 0), but beyond ≈ 40 Myrs, the Precambrian fluctuations slowly decrease with scale with *H* ≈ -0.2 whereas for Δ*t* ≈> 3 Myrs, the Phanerozoic has *H* ≈ 0.1. Further evidence for similar Phanerozoic and Precambrian statistics up to Δ*t* ≈ 40 Myrs, but divergent at larger Δ*t,* is displayed by the RMS δ^18^*O* statistics (fig. S4). Whereas in the Phanerozoic, the *H* ≈ 0.3 scaling continues to ≈ 500 Myrs, over the same range, the Precambrian has *H* ≈ -0.15. Since *H* > 0 is associated with unstable “wandering” behaviour and *H* < 0 with stable converging behaviour, we see that for Δ*t* ≈> 40 Myrs, the Precambrian is overall more stable than the Phanerozoic. Finally, for Δ*t* ≈>500Myrs the comparison of the red in fig. S4 (the full range) and blue (Precambrian) analyses indicate that the main 500 Myr variability occurs in the Phanerozoic, and that in the Precambrian there is significantly less variability until Gyr scales or longer (the extreme limit of the full data set (red, fig. S4), at Δ*t* > 600 Myr seem to indicate an overall Precambrian stabilization).

Information about the clustering and sparseness of the ρ(*t*) spikes can be gleaned for the remaining multifractal exponents *C*_1_, α. Fig. S5 shows the *F*(Δ*t*) whose logarithmic slope is the intermittency exponent *C*_1_ (there is also a *C*_1_ for the δ^18^*O* values, but these are fairly small (≈0.03) and were not considered further). Interestingly, with the exception of the small Δ*t* part of the Precambrian data, *C*_1_ ≈ 0.2 for both the Phanerozoic and Precambrian (similar to most of the other geochronologies, fig. 3 middle). Fig. S6 shows the estimates of α, the index of multifractality. Recall that 0≤α≤2 (the monofractal to lognormal multifractal limits) and as with the α for the other chronologies (fig. S8) there is a rough trend – to rise from small α to values ≈ 1.5 for larger Δ*t*.

Finally, fig. S7 shows the probability distribution of the inter-measurement time intervals Dt, associated with the length Sadler effect, the largest Dt being 325 Myrs. We see that for the probability of the extremes, *q_D_* ≈ 1.54 which is low (implying an infinite variance since *q_D_*<2), but quite comparable to other values (compare with fig. 4).

Fig. S1: The 25399 points of the Isson ^18^O Carbonate assemblage on a time axis that was discretized at 100kyr resolution so that there are 35040 intervals covering the last 3.504 Gyrs (the oldest sample). The number per interval is shown in blue (i.e. densities per 100 kyr). We see that for example the maximum density is ≈ 6503/Myr that occurred 650.3 Myr ago). The red points are the δ^18^O Carbonate values multiplied by 20 so as to be clear when presented on the same axes.

Fig. S2: A plot of the logarithm of the age difference between successive samples (Dt) and the logarithm of the age (*t*) for the two assemblages discussed in the text: black is the Isson ^18^O carbonates^1^ (fig. S1) and the red is the Grossman paleotemperatures^2^ (Fig. 1). Only nonzero differences were plotted; for the Isson assemblage these represented 13536 out of a total of 25399 measurements (53%). However this hides a large difference between the Phanerozoic (<600Myr) and Precambrian (>600Myrs): in the former, 8942 (out 20700, 43%) represent 100 kyr bins in which two or more measurements were taken whereas for the Precambrian, 4594 out of 4700 (98%) were taken at the same bin as at least one other (i.e. with Dt = 0). In comparison, the Grossman chronologies had Dt = 0 for 7925 out of 11874 measurements (67%).

This plot can now be used to estimate the resolutions of the measurements estimated as the smallest nonzero interval Dt. Consider for the moment, the Grossman (red points) that were reported at 10kyr resolution (the upper horizontal dashed line). Although we see that – due precisely to the clustering of measurements discussed in this text - there is a considerable spread in Dt yet, the 10 kyr minimum Dt appears to be respected throughout the age range. However, the situation with the Isson chronology is different: up to nearly 100 Myrs ago, the minimum Dt was about 100 years but then, going back further in time, it appears to be ill - defined although earlier - in the Precambrian it is exactly 1 Myr. This drastic change in the resolution with age makes this series much less homogeneous that the others considered here; in the analyses in Figs. S3 - 6, we chose a compromise and discretized the time axis at 100 kyrs resolution.

Fig. S3: A comparison of the mean, normalized measurement density fluctuations discretized at 100 kyr resolution for the Phanerozoic (brown, estimated from the data back to 600 Myrs), the full range (0 – 3504 Myrs, red, corresponding to the blue in fig. 3) and just the Precambrian (approximated by the period 600 – 3504 Myrs, in blue). The actual nominal densities varied greatly according to the age (fig. S2), the mean densities used for normalizing each of the above curves were 34.5/Myrs (<600Myrs), 1.6/Myrs (600 - 3504 Myrs), 7.4/Myrs (0 - 3504 Myrs). For the analyses in this figure, the densities were first estimated on a time axis discretized to 100 kyr resolution.

Fig. S4: The RMS δ^18^O values shown for the Phanerozoic (20700 values, brown), the Precambrian (600- 3504 Myrs, blue, 4699 values) and the full data set (0 - 3504 Myrs, red, 25399 values, same as in fig. 2 but without the multiplication by 4 to roughly calibrate it in terms of temperatures). Note that the extreme large Δ*t* red fluctuation is not visible since it is hidden under the extreme blue one. The Phanerozoic part therefore has an average density of 20700/600 = 34.5/Myrs whereas the later part has a density of 4.62/Myr, i.e. it is on average 21 times less dense and will hence dominate the shorter time scale statistics (indeed, the brown and red overlap quite closely over their entire common range). In contrast the Precambrian (blue) analysis shows about the same tendency up to about 40 Myrs (see the reference lines with slopes 0.3), but at larger Δ*t* declines slowly with *H* ≈ -0.15.

Fig. S5: A comparison of the *F*(Δ*t*) curves whose logarithmic slope is the intermittency exponent *C*_1_ (eq. M5). For the top (Phanerozoic), we find *C*_1_ ≈ 0.2 (close to the reference lines) over the whole range of time scales, similar to the other geochronologies (e.g. fig. 2 middle). However, the full series (red) and the Precambrian (blue) has *C*_1_ ≈ 0.2 for Δ*t* ≈>10 Myrs. Indeed, for Δ*t* ≈ < 10 Myrs the Precambrian (blue) have a slope *C*_1_ ≈ 1 (bottom dashed reference line), this is the theoretical maximum for process in 1-D (i.e. on the time axis). It indicates extreme intermittency, here presumably an artefact of the existence of only a very small number of very sparse “spikes” at these short time lags (recall from fig. S2 that for most of the pre Phanerozoic chronology, the resolution is 1 Myrs so that the statistics are poor until 10 Myrs or so).

Fig. S6: Estimates of the multifractal index α for the Phanerozoic (brown) Precambrian (blue) and the full range (red). The Phanerozoic has a fairly constant index α ≈ 1.5 up to about 100 Myrs. In comparison the Precambrian has low α for small lags but increases to α ≈ 1.5 after about the same (100 Myrs) time scale.

Fig. S7: A log- log plot of the probability distribution of a inter measurement intervals Dt exceeding a fixed threshold. As indicated in fig. S2, out of the total number of measurements, 53% had Dt = 0. This is the distribution of the 11864 distinct (Dt > 0) measurement intervals. Recall that *q_D_* is the exponent governing the length Sadler effect, and compare this to fig. 4.

# Part 2: Full analyses

In the main body of the paper, only a fraction of our analyses were presented: analyses representative of different scale ranges and data types, and this, in order to facilitate their comparison, to highlight their commonalities. In this section of the supplement, we present analyses of the full ensemble of geochronologies.

## Estimates of $\alpha$ for datasets in the main text


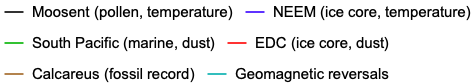


Macroclimate

Megaclimate

Climatee

Macroweather

Figure S8: Estimates for the multifractal index $\alpha$ as a function of timescale for all of the representative datasets analysed in the main body of the report. The transition scales between different climate regimes are shown by vertical grey bars. At short time scales, $\alpha\approx0$, but it undergoes a rapid increase, often at the same timescale at which the scaling regime changes, to reach a plateau of $1\lesssim\alpha\lesssim1.5$ at longer timescales. This shows that at short timescales, the measurement density approaches a monofractal set, whereas at longer timescales it appears more as a multifractal.

## Holocene analyses


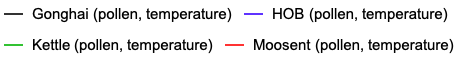


Macroweather

Climate


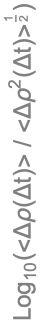


Figure S9: The results of Haar analysis on datasets that analysed fossilized pollen records over the Holocene. a) $<\rho\left( \Delta t \right)>$, the slope of which gives an estimate of $H$, b) plot of log $F\left( \Delta t \right)$, the slope of which gives an estimate of $C_{1}$ (and which differs from the log-log plot of the ratio between the fluctuation in measurement density and its root mean square by a constant coefficient for constant $\alpha\text{- shown on the right axis})$, and c) the correlation coefficient between fluctuations in measurement density and fluctuations in the measured quantity. In grey in plot a) are the fluctuations in temperature for the EDC (left) and Grossman (right) datasets to demonstrate the different scaling regimes of the climate system. The transition scale between different climate regimes is shown by a vertical grey bar. Two distinct scaling regimes can be seen: one with $H\approx-0.5$ and $C_{1}\approx0$ (fluctuating sedimentation rate dominant) and a second with $H\approx0.5$ and $C_{1}\approx0$.2 (erosion process dominant), with a transition timescale around 300 yr.

Climate

Macroweather

Figure S10: Estimates for the multifractal index $\alpha$ as a function of timescale for datasets that analysed fossilized pollen records over the Holocene. The transition timescale between different climate regimes is shown by a vertical grey bar. At short timescales, $\alpha\approx0$, but it undergoes a rapid increase - at the same timescale at which the scaling regime changes - to reach a plateau of $1\lesssim\alpha\lesssim1.5$ at longer timescales. This shows that at short timescales, the measurement density approaches a monofractal set, whereas at longer timescales it appears more as a multifractal. $\alpha$ for the Moosent dataset does appear to decrease steadily after an initial peak at the transition timescale, however $\alpha$ remains much larger than at the very shortest timescales.


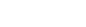


Figure S11: the probability distribution $Pr(\Delta\tau'>\Delta\tau)$ of intervals between consecutive datapoints in the paleo chronologies of datasets that analysed fossilized pollen records over the Holocene, where $\Delta\tau'$ is the duration of a random interval and $\Delta\tau$ is a threshold. Black dotted lines show the best fit Gaussian distribution, whereas straight black lines show a best fit linear curve on a portion of the data. The exponents $q_{D}$ are shown for the linear log-log fits, where $Pr\approx\Delta\tau^{-q_{D}}$. Distributions have been offset vertically for presentation. A Gaussian fit does not describe the data sets well, and that all the data sets have long linear tails demonstrating extreme scaling behaviour in the probability distribution, particularly for the HOB dataset with $q_{D}=1.2$.

**
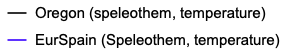
**

Macroweather

Climate


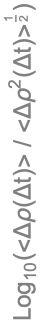


Figure S12: The results of Haar analysis on datasets that analysed speleothems over the Holocene. a) $<\rho\left( \Delta t \right)>$, the slope of which gives an estimate of $H$, b) plot of log $F\left( \Delta t \right)$, the slope of which gives an estimate of $C_{1}$, and c) the correlation coefficient between fluctuations in measurement density and fluctuations in the measured quantity. In grey in plot a) are the fluctuations in temperature for the EDC (left) and Grossman (right) datasets to demonstrate the different scaling regimes of the climate system. The transition scale between different climate regimes is shown by a vertical grey bar. Two distinct scaling regimes can be seen: one with $H\approx-0.5$ and $C_{1}\approx0$ (fluctuating sedimentation rate dominant) and a second with $H\approx0.33$ and $C_{1}\approx0$.3 (erosion process dominant), with a transition timescale or around 100yr.

Climate

Macroweather

Figure S13: Estimates for the multifractal index $\alpha$ as a function of timescale for datasets that analysed speleothems over the Holocene. The transition timescale between different climate regimes is shown by a vertical grey bar. At short timescales, $\alpha\approx0$, but it undergoes a rapid increase - at the same timescale at which the scaling regime changes - to reach a plateau of $1\lesssim\alpha\lesssim1.5$ at longer timescales. This shows that at short timescales, the measurement density approaches a monofractal set, whereas at longer timescales it appears more as a multifractal.


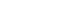


Figure S14: the probability distribution $Pr(\Delta\tau'>\Delta\tau)$ of intervals between consecutive datapoints in the paleo chronologies of datasets that analysed speleothems over the Holocene, where $\Delta\tau'$ is the duration of a random interval and $\Delta\tau$ is a threshold. Black dotted lines show the best fit Gaussian distribution, whereas straight black lines show a best fit linear curve on a portion of the data. The exponents $q_{D}$ are shown for the linear log-log fits, where $Pr\approx\Delta\tau^{-q_{D}}$. Distributions have been offset vertically for presentation. A Gaussian fit does not describe the EurSpain data set well, although it describes the Oregon dataset passably at the beginning. Both data sets have linear tails demonstrating scaling behaviour in the probability distribution, although the scaling behaviour of the EurSpain dataset is considerably more extreme than that seen in Oregon.

## Quaternary analyses


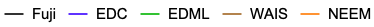


Climate

Macroweather

Macroclimate


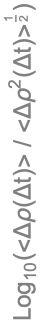


Figure S15: The results of Haar analysis on ice core datasets that measured temperature over the Quaternary. a) $<\rho\left( \Delta t \right)>$, the slope of which gives an estimate of $H$, b) plot of log $F\left( \Delta t \right)$, the slope of which gives an estimate of $C_{1}$, and c) the correlation coefficient between fluctuations in measurement density and fluctuations in the measured quantity. In grey in plot a) are the fluctuations in temperature for the EDC (left) and Grossman (right) datasets to demonstrate the different scaling regimes of the climate system. The transition scales between different climate regimes are shown by vertical grey bars. Two distinct scaling regimes can be seen for each dataset: one with $H\approx-0.5$ and $C_{1}\approx0$ (fluctuating sedimentation rate dominant) and a second with $H>0$ and $C_{1}>0$ (erosion process dominant). There are different transition timescales for the datasets that depend on their latitude. The datasets from the Northern polar regions (WAIS and NEEM) transition at around 100 yr, whereas the datasets from the Southern polar regions (EDC and EDML) transition around 1000 yr. The dataset from mid latitudes (Fuji) transitions at around 10,000 yr.

Macroweather

Climate

Macroclimate

Figure S16: Estimates for the multifractal index $\alpha$ as a function of timescale for ice core datasets that measured temperature over the Quaternary. The transition scales between different climate regimes are shown by vertical grey bars. At short timescales, $\alpha\approx0$, but it undergoes a rapid increase - at the same timescale at which the scaling regime changes - to reach a plateau of $1\lesssim\alpha\lesssim1.5$ at longer timescales. This shows that at short timescales, the measurement density approaches a monofractal set, whereas at longer timescales it appears more as a multifractal. The Fuji dataset does not seem to follow this trend well, as its value for $\alpha$ reaches a peak before decreasing again to values similar to those seen at very high frequencies.


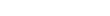


Figure S17: the probability distribution $Pr(\Delta\tau'>\Delta\tau)$ of intervals between consecutive datapoints in the paleo chronologies of datasets that measured temperature over the Quaternary, where $\Delta\tau'$ is the duration of a random interval and $\Delta\tau$ is a threshold. Black dotted lines show the best fit Gaussian distribution, whereas straight black lines show a best fit linear curve on a portion of the data. The exponents $q_{D}$ are shown for the linear log-log fits, where $Pr\approx\Delta\tau^{-q_{D}}$. Distributions have been offset vertically for presentation. A Gaussian fit does not describe most of the datasets well, except for the WAIS dataset which roughly follows a Gaussian distribution until the very longest gaps. All the datasets have linear tails demonstrating scaling behaviour in the probability distribution. In particular, the NEEM dataset displays very extreme scaling with $q_{D}=0.6$. It should be noted there is no useful probability distribution for the Fuji dataset as it was sampled in such a way that all the times allocated to only had gaps of either 250 yr or 500 yr between them. This means that the measurement density was still free to fluctuate, but the probability distribution only consisted of two values, hence it was not included in this plot.

Macroweather

Macroclimate

Climate


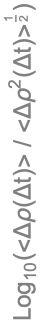


Figure S18: The results of Haar analysis on the Vostok dataset, an ice core that measured temperature anomaly over the Quaternary. a) $<\rho\left( \Delta t \right)>$, the slope of which gives an estimate of $H$, b) plot of log $F\left( \Delta t \right)$, the slope of which gives an estimate of $C_{1}$, and c) the correlation coefficient between fluctuations in measurement density and fluctuations in the measured quantity. In grey in plot a) are the fluctuations in temperature for the EDC (left) and Grossman (right) datasets to demonstrate the different scaling regimes of the climate system. The transition scales between different climate regimes are shown by vertical grey bars. Two distinct scaling regimes can be seen: one with $H\approx-0.5$ and $C_{1}\approx0$ (fluctuating sedimentation rate dominant) and a second with $H\approx0.5$ and $C_{1}\approx0.4$ (erosion process dominant). The transition timescale occurs around 3000 yr.

Macroweather

Climate

Macroclimate

Figure S19: Estimates for the multifractal index $\alpha$ as a function of timescale for the Vostok ice core dataset that measured temperature anomalies over the Quaternary. The transition scales between different climate regimes are shown by vertical grey bars. At short timescales, $\alpha\approx0$, but it undergoes a rapid increase - at the same timescale at which the scaling regime changes - to reach a plateau of $\alpha\approx1.4$ at longer timescales. This shows that at short timescales, the measurement density approaches a monofractal set, whereas at longer timescales it appears more as a multifractal.

Figure S20: the probability distribution $Pr(\Delta\tau'>\Delta\tau)$ of intervals between consecutive datapoints in the Vostok ice core paleo chronology that measured temperature anomaly over the Quaternary, where $\Delta\tau'$ is the duration of a random interval and $\Delta\tau$ is a threshold. The black dotted line shows the best fit Gaussian distribution, whereas the straight black line shows a best fit linear curve on a portion of the data. The exponent $q_{D}$ is shown for the linear log-log fit, where $Pr\approx\Delta\tau^{-q_{D}}$. A Gaussian fit does not describe the dataset well. The dataset has a linear tail with $q_{D}=23.9$ demonstrating some scaling behaviour in the probability distribution.

**
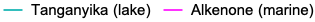
**

Macroweather

Climate

Macroclimate


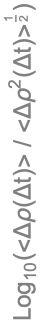


Figure S21: The results of Haar analysis on lake and marine sediment datasets that measured temperature over the Quaternary. a) $<\rho\left( \Delta t \right)>$, the slope of which gives an estimate of $H$, b) plot of log $F\left( \Delta t \right)$, the slope of which gives an estimate of $C_{1}$, and c) the correlation coefficient between fluctuations in measurement density and fluctuations in the measured quantity. In grey in plot a) are the fluctuations in temperature for the EDC (left) and Grossman (right) datasets to demonstrate the different scaling regimes of the climate system. The transition scales between different climate regimes are shown by vertical grey bars. Just one scaling regime can be seen for each dataset with $H\approx-0.1$ and $C_{1}\approx0.1.$ This indicates considerable intermittency that is a result of strong erosion processes, yet the measurement density still approaches a well-defined mean regardless.

Macroweather

Climate

Figure S22: Estimates for the multifractal index $\alpha$ as a function of timescale for marine sediment datasets that measured temperature over the Quaternary. Transition scales between different climate regimes are shown by vertical grey bars. These datasets did not transition between scaling regimes, and no change in behaviour for $\alpha$ is observed. Instead, $\alpha$ is seen to steadily increase for all timescales.

Figure S23: the probability distribution $Pr(\Delta\tau'>\Delta\tau)$ of intervals between consecutive datapoints in paleo chronologies from lake and marine sediment datasets that measured temperature over the Quaternary, where $\Delta\tau'$ is the duration of a random interval and $\Delta\tau$ is a threshold. Black dotted lines show the best fit Gaussian distribution, whereas straight black lines show a best fit linear curve on a portion of the data. The exponents $q_{D}$ are shown for the linear log-log fits, where $Pr\approx\Delta\tau^{-q_{D}}$. Distributions have been offset vertically for presentation. A Gaussian fit does not describe either dataset well and both datasets have linear tails demonstrating scaling behaviour in the probability distribution.

**
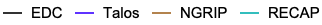
**

Macroclimate

Macroweather

Climate


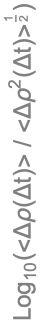


Figure S24: The results of Haar analysis on ice core datasets that measured dust concentrations over the Quaternary. a) $<\rho\left( \Delta t \right)>$, the slope of which gives an estimate of $H$, b) plot of log $F\left( \Delta t \right)$, the slope of which gives an estimate of $C_{1}$, and c) the correlation coefficient between fluctuations in measurement density and fluctuations in the measured quantity. In grey in plot a) are the fluctuations in temperature for the EDC (left) and Grossman (right) datasets to demonstrate the different scaling regimes of the climate system. The transition scales between different climate regimes are shown by vertical grey bars. Two distinct scaling regimes can be seen for each dataset: one with $H\approx-0.5$ and $C_{1}\approx0$ (fluctuating sedimentation rate dominant) and a second with $H\approx0.3$ and $C_{1}\approx0.2$ (erosion process dominant). The transition timescale appears to be around 300 yr.

Climate

Macroclimate

Macroweather

Figure S25: Estimates for the multifractal index $\alpha$ as a function of timescale for ice core datasets that measured dust concentrations over the Quaternary. The transition scales between different climate regimes are shown by vertical grey bars. At short timescales, $\alpha\approx0$, but it undergoes a rapid increase - at the same timescale at which the scaling regime changes - to reach a plateau of $1\lesssim\alpha\lesssim1.5$ at longer timescales. This shows that at short timescales, the measurement density approaches a monofractal set, whereas at longer timescales it appears more as a multifractal. $\alpha$ for the NGRIP dataset does appear to decrease steadily after an initial peak at the transition timescale, however $\alpha$ remains much larger than at the very shortest timescales. This suggests that $\alpha$ does not remain in the range $1\lesssim\alpha\lesssim1.5$ indefinitely.


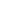


Figure S26: the probability distribution $Pr(\Delta\tau'>\Delta\tau)$ of intervals between consecutive datapoints in paleo chronologies from ice core datasets that measured dust concentration over the Quaternary, where $\Delta\tau'$ is the duration of a random interval and $\Delta\tau$ is a threshold. Black dotted lines show the best fit Gaussian distribution, whereas straight black lines show a best fit linear curve on a portion of the data. The exponents $q_{D}$ are shown for the linear log-log fits, where $Pr\approx\Delta\tau^{-q_{D}}$. Distributions have been offset vertically for presentation. A Gaussian fit does not describe any dataset well, except for the start of the Talos distribution. However, all datasets have long linear tails, demonstrating scaling behaviour in the probability distribution.

**
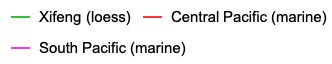
**

Macroweather

Climate

Macroclimate


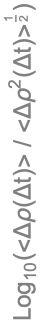


Figure S27: The results of Haar analysis on loess and marine sediment datasets that measured dust concentrations over the Quaternary. a) $<\rho\left( \Delta t \right)>$, the slope of which gives an estimate of $H$, b) plot of log $F\left( \Delta t \right)$, the slope of which gives an estimate of $C_{1}$, and c) the correlation coefficient between fluctuations in measurement density and fluctuations in the measured quantity. In grey in plot a) are the fluctuations in temperature for the EDC (left) and Grossman (right) datasets to demonstrate the different scaling regimes of the climate system. The transition scales between different climate regimes are shown by vertical grey bars. Two distinct scaling regimes can be seen for each dataset: one with $H\approx-0.5$ meaning that the measurement density is converging to a well-defined mean and a second with $H\approx0.4$ meaning there is no well-defined sampling rate. The transition timescale between the two regimes is about 3000 yr. However, the scaling regimes are much less clear in the plots in b). Only the scaling regimes for the South Pacific dataset are clear in b) where $C_{1}\approx0$ in the first and $C_{1}\approx0.2$ in the second. However, for the other two datasets, $C_{1}\approx0.2$ at all time scales, suggesting that erosion processes were significant at all timescales for these datasets.

Macroclimate

Climate

Figure S28: Estimates for the multifractal index $\alpha$ as a function of timescale for loess and marine sediment datasets that measured dust concentrations over the Quaternary. The transition scales between different climate regimes are shown by vertical grey bars. At short timescales, $\alpha\approx0$, but it undergoes a rapid increase - at the same timescale at which the scaling regime changes - to reach a plateau of $1\lesssim\alpha\lesssim1.5$ at longer timescales. This shows that at short timescales, the measurement density approaches a monofractal set, whereas at longer timescales it appears more as a multifractal.


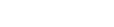


Figure S29: the probability distribution $Pr(\Delta\tau'>\Delta\tau)$ of intervals between consecutive datapoints in paleo chronologies from loess and marine sediment datasets that measured dust concentration over the Quaternary, where $\Delta\tau'$ is the duration of a random interval and $\Delta\tau$ is a threshold. Black dotted lines show the best fit Gaussian distribution, whereas straight black lines show a best fit linear curve on a portion of the data. The exponents $q_{D}$ are shown for the linear log-log fits, where $Pr\approx\Delta\tau^{-q_{D}}$. Distributions have been offset vertically for presentation. A Gaussian fit describes the beginning of the Xifeng and Central Pacific datasets well, but all datasets have long linear tails for larger gaps, demonstrating scaling behaviour in the probability distribution.
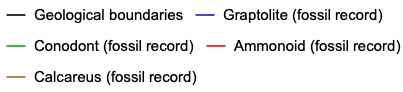


## Removal of large timescale trends

The statistical analyses estimate fluctuations at a given lag Δ*t* by averaging over all the disjoint intervals of length Δ*t*, the underlying assumption being that the series are statistically stationary. While this is a reasonable approximation for many of the series ti clearly is problematic for ice cores where even though (snow) sedimentation rates may be stationary, the lower parts of the core are significantly compressed due to the weight of the overlying ice. In this case, the series are clearly not stationary, yet it is straightforward to largely eliminate the effect by removing an overall linear or quadratic trend. Fig. S30 shows the result of this on the EDC series. Since the compression is a low frequency effect, as expected, the short lag statistics are essentially unaffected and the qualitative and even quantitative conclusions are essentially unchanged.


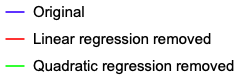


Figure S30: The measurement density of the EDC dataset, an ice ore that measured dust concentrations over the Quaternary as a function of the age of the sediment, with the temporal resolution degraded by a factor of 100 (top) and $<\rho\left( \Delta t \right)>$, the slope of which gives an estimate of $H$ (bottom). These plots are shown with the original measurement densities taken from the dataset (blue), the measurement densities with a linear regression removed (red), and with a quadratic regression removed (green). This was done to simulate the compression that the sediment undergoes over time due to pressure. By removing this compressive factor, it is possible to see that $<\rho\left( \Delta t \right)>$ is not strongly affected by this process except at the very lowest frequencies. Therefore, the compression of sediments does not seem to affect the conclusions drawn in this paper about the scaling regimes.

## Supplementary References

1 Isson, T. & Rauzi, S. Oxygen isotope ensemble reveals Earth’s seawater, temperature, and carbon cycle history *Science* **383**, 666–670 (2024).

2 Grossman, E. L. & Joachimski, M. M. Ocean temperatures through the Phanerozoic reassessed. *Scientific Reports* **12**, 8938 (2022). <https://doi.org/https://doi.org/10.1038/s41598-022-11493-1>
